# Variable and Variant Protein Multigene Families in *Babesia bovis* Persistence

**DOI:** 10.3390/pathogens8020076

**Published:** 2019-06-11

**Authors:** David R. Allred

**Affiliations:** 1 Department of Infectious Diseases and Immunology, University of Florida, Gainesville, FL 32611, USA; allredd@ufl.edu; Tel.: +1-352-294-4126; 2 Emerging Pathogens Institute, University of Florida, Gainesville, FL 32611, USA; 3 Genetics Institute, University of Florida, Gainesville, FL 32611, USA

**Keywords:** *Babesia*, persistent infection, antigenic variation, cytoadhesion, immunodominance, immune evasion

## Abstract

Cattle infected with *Babesia bovis* face a bifurcated fate: Either die of the severe acute infection, or survive and carry for many years a highly persistent but generally asymptomatic infection. In this review, the author describes known and potential contributions of three variable or highly variant multigene-encoded families of proteins to persistence in the bovine host, and the mechanisms by which variability arises among these families. Ramifications arising from this variability are discussed.

## 1. Introduction

Although pathogens sometimes kill their host, in the long run parasites do not benefit from this outcome. The death of the host reduces dramatically their opportunity for pickup by a vector and transmission to a new host [1]. Significantly, *Rhipicephalus microplus* and *Rhipicephalus annulatus*, the major tick vectors responsible for transmission of the hemoparasite, *Babesia bovis*, remain attached to the mammalian host for three weeks or more during natural transmission [2]. Over comparable time periods, steers infected experimentally with *B. bovis* experienced parasite populations that underwent expansions and contractions of several orders magnitude [3]. At times, levels of parasitemia rose high enough that parasites could be identified easily by microscopy and likely could easily have been picked up by a tick vector for biological transmission. Although not directly demonstrated, it is reasonable to expect that vast fluctuations similarly occur under conditions of natural infection. The slow pace by which the attached tick vector feeds and completes its own life cycle to some extent mitigates the effects of these fluctuations in parasite population density on transmission. Not all the factors responsible for creating these dynamic population swings are known or understood. One generally accepted assumption is that they are a result of interplay between the host immune system and variable components of the parasite. The idea is that, immune recognition of an essential function leads to killing or suppression of the parasite population, whereas variability or variation in the parasite component responsible for that essential function allows escape from immune pressure, leading to population expansion. We will explore the nature of this interplay from the perspective of parasite multigene protein families and present possibilities for how this interplay leads to parasite persistence.

## 2. Major Variable and Variant Multigene Protein Families

Prior to acquisition of the *B. bovis* genome in 2007 [4], multiple variable and variant multigene protein families had already been identified, primarily during efforts to identify putative vaccine candidates. This was facilitated by the development of monoclonal antibody (mAb) technology that enabled rapid identification of parasite proteins and the sequences encoding them. In this way, rhoptry-associated protein-1 (RAP1) and merozoite surface antigen-1 (MSA1) were identified, each of which was found to be variable in structure and encoded by multiple genes of the *rap1* and *msa1* families, respectively [5,6,7,8,9]. Similarly, mAbs identified a large 225 KDa protein associated with the cytoplasmic surface of the *B. bovis*-infected erythrocyte (BbIE) membrane that turned out to represent the spherical body protein-2 (SBP2) family, a small family of proteins associated with protein trafficking [10,11,12]. The use of bovine infection sera reactive with the surface of BbIE identified an isolate-specific heterodimeric parasite-derived protein complex [13] of 104–120 KDa. The polypeptides of this complex were later found to vary rapidly during infection with a clonal parasite line, demonstrating their involvement in rapid antigenic variation, and earning the name “Variant Erythrocyte Surface Antigen- 1” (VESA1) [14]. Subsequent development of mAbs reactive with VESA1 confirmed its involvement in antigenic variation and enabled identification of the large *ves* multigene family encoding the variant protein [15].

With the acquisition of the *B. bovis* T2Bo genome [4] it became possible to mine the predicted proteome for additional protein multigene families. This resource provided clarification of the overall diversity of the various gene families that had already been identified. A surprise found hiding within the *B. bovis* genome was a relatively large multigene family comprised of small open reading frames, resulting in the fitting name *smorf*. Genes of this family were found to be dispersed among and often juxtaposed with members of the *ves* family, which is itself scattered across the entire genome in small clusters [4]. Like the *ves* family, *smorf* genes were found to be both expressed and variant among isolates [16]. Moreover, by searching the sequence database the “6-cys” family of proteins, which in *Plasmodium falciparum* is important to sexual development in the mosquito vector, was identified [17,18,19]. While lacking the complexity of higher eukaryotes, *B. bovis* has undergone selection and expansion of multigene protein families to serve the needs of its highly specialized life-cycle. I will attempt to summarize here the structures of the various proteins and protein families, and to define what makes them so useful to the parasite in its survival and persistence. The focus will be upon proteins with apparent functions in direct host-pathogen interactions, and not on amplified gene families for proteins serving, for example, structural roles.

### 2.1. “Variable” vs. “Variant” Proteins

A distinction needs to be made at this point between “variable” and “variant” proteins. Both categories of protein families may contribute to persistence, but are likely to do so in different ways. Because of this, the nature of the selective forces acting upon each class of proteins will differ. For the purposes of this review, a “variable” multigene protein family is defined as one containing two or more members, each of which differs in sequence, which recombine with one another infrequently if at all, are stable in sequence during the course of an infection within an individual host, and may be co-expressed within an individual parasite. A “variant” multigene protein family is considered one with the following characteristics: [i] the family contains more than several members, [ii] each member differs in sequence, [iii] the family is generally expressed monoparalogously or monoallelically, [iv] the expressed copy may recombine with others as an integral aspect of the family’s biology, and [v] the expressed member is routinely altered in sequence during the course of an infection within an individual host. This is essentially a slight broadening of the definition provided by Borst [20]. These distinctions are summarized in Table 1. As this review is focused on *B. bovis*, specific examples from this parasite will be discussed, although the principles hold for other organisms as well. Among those families mentioned above, the *rap1*, *msa1*, *sbp2*, and *6-cys* multigene families and their encoded proteins fall into the variable category. The diversity present in variable genes coupled with their relative stability make them good markers for use in epidemiologic studies at the isolate level, and advantage has been taken of *rap1* and *msa1* sequences repeatedly for this purpose (for example, see [21,22,23,24,25,26,27,28,29,30]). In contrast, members of the *ves* multigene family are highly variant in sequence and the expressed member(s) further vary rapidly during the course of infection in an individual host [14]. As a result, although transcribed monoparalogously, the proteins encoded by this family change swiftly and dramatically during infection [15,31]. The full extent and dynamics of variation among *smorf* genes is not yet clear, and it seems that this family may possess characteristics of both classes [16]. The *rap1*, *ves*, and *smorf* gene families will be discussed individually as examples of the various multigene family behaviors, the structures of their encoded proteins, and known or potential roles they may play in host–pathogen interactions.

### 2.2. Variable Proteins—RAP-1

**Figure 1:**
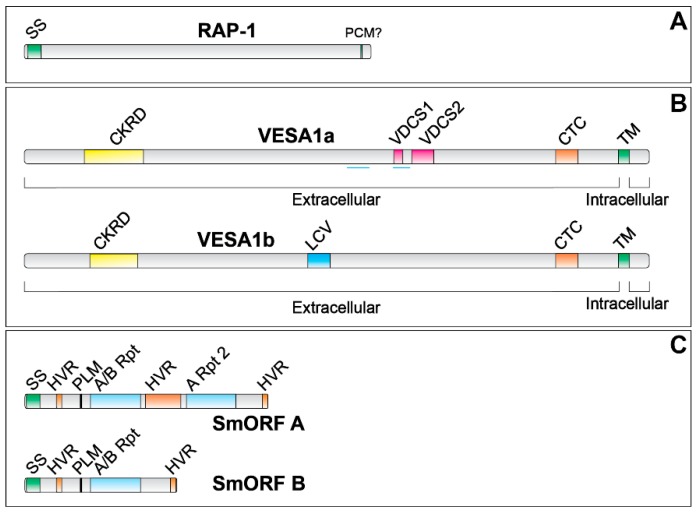
Comparative structures of RAP-1, VESA1, and SmORF proteins. Comparative structures and significant domains of stereotypic examples of each of the protein families discussed in this review is provided here. (**A**) RAP-1 proteins have an N-terminal signal sequence (SS) for entrance into the endoplasmic reticulum and may have a site for post-translational covalent modification (PCM) that results in membrane anchoring. These proteins partition with integral membrane proteins but do not possess a transmembrane domain [6]. (**B**) VESA1 proteins do not possess an N-terminal signal sequence, but do have a C-terminal transmembrane domain (TM). VESA1a contains a cysteine-lysine-rich domain (CKRD), variable domain conserved sequences (VDCS), and a C-terminal cysteine-rich domain (CTC). VESA1a frequently contains coiled-coil sequences (thin blue lines beneath the VDCS domain). VESA1b possesses a CKRD, CTC, and TM domain but no VDCS domain. On the other hand, they contain a centrally located low-complexity variant domain (LCV) [15,31,55]. (**C**) SmORF proteins possess an SS domain for entry into the endoplasmic reticulum, and hypervariable regions (HVR) in the N-terminal third and near the C-terminus. Type A and B contain a central repeat domain (A/B Rpt), whereas type B possess an additional HVR and a second (or more) repeat region (A Rpt) [16]. Proteins are depicted roughly to scale relative to one another. SmORF proteins additionally possess a PEXEL-like motif not found in the other two families that is proteolytically processed as a part of trafficking and proper staging.

RAP-1 (for rhoptry-associated protein-1) proteins (Figure 1A) are so named because of their localization to the rhoptry organellar compartment found in the anterior half of apicomplexan parasites. As components of the rhoptries, these roughly 60 kDa proteins have been suggested to participate in the invasion of host erythrocytes by both merozoite and sporozoite stages [32,33,34,35]. Consistent with this, BdRAP-1 has been shown to bind as a soluble protein to a non-proteinaceous component of host erythrocytes [36]. No previously characterized functional domains are recognizable in this protein family to suggest an explanation for their binding behavior. The structure of RAP-1 proteins includes a predicted N-terminal signal sequence for entry into the endoplasmic reticulum, but lacks a proteolytically processed *Plasmodium* export element (PEXEL)-like motif for trafficking to or retention within the spherical body compartment [37]. Early work on subcellular localization of RAP-1 proteins suggested that it is soluble, based upon partitioning properties in Triton X-114 [6]. By analogy with related proteins in *Plasmodium*, the protein is expected to be stored within the rhoptry compartment. However, in different studies immunolocalization has suggested various compartments, from clear localization in the rhoptries (*B. divergens*) [36] to diffuse intracellular staining of parasites (*B. gibsoni*) [38], to secretion into and association with the infected erythrocyte membrane (*B. bovis*) [35]. Although possible, it is unlikely that these three species, all of which are members of *Babesia* sensu stricto, localize RAP-1 in different compartments. A more likely explanation has to do with technical differences in the nature and efficacy of cellular fixation and stabilization of antigen distribution employed in the studies. Assuming a rhoptry localization, the nature of the trafficking signal (or other mechanism) resulting in this localization is unknown. Based upon metabolic incorporation of [^3^H]glucosamine this protein is glycosylated [6], consistent with a function in the extracellular environment and passage through a golgi-like compartment.

RAP-1 is immunodominant based upon elicitation of antibodies during infection [6]. B-cell epitopes were detected in RAP-1 proteins early on that were shared among isolates and even between *B. bovis* and *B. bigemina*, suggesting this protein’s potential utility in vaccine development despite its clear genetic polymorphism [34,39,40,41,42]. However, those sequences that are conserved between *B. bovis* and *B. bigemina* are poorly immunogenic, and antibodies raised against one species do not react with native protein of the opposite species [43]. The ultimate significance of this to vaccine development is debatable, as evidence for the ability to inhibit erythrocyte invasion by antibodies to RAP-1 proteins is equivocal. Although RAP-1 proteins are immunodominant [6] and the monoclonal antibody to a B-cell epitope of *B. bigemina* RAP-1 can neutralize erythrocyte invasion in vitro [44], antibody titers to the same epitope, generated in vivo by prior infection, had essentially no correlation with protection [32]. Similarly, no evidence was found for inhibition of erythrocyte invasion by antibodies to BdRAP-1 (i.e., from *B. divergens*), even at a level of 2 mg ml^-1^ [36]. Moreover, immunization of cattle with recombinant BbRAP-1 under conditions that elicited significant T-helper IFN-γ and IgG responses, both of which were considered important metrics of immunity to achieve during vaccination, failed to provide protection from virulent *B. bovis* challenge [45]. This was the case despite the immunodominance of RAP-1 proteins and shared T-cell epitopes among different geographic isolates [46]. RAP-1 is a sobering example of the irrelevance of overt immunodominance as a criterion in choosing vaccine candidates.

Perhaps the biggest surprise in the failure of RAP-1 to provide protection was that, coupled with conserved T-cell epitopes, there is limited within-species diversity to this protein family, even though diversity among species may be high. In *B. bovis* and *B. canis*, the *rap1* locus is comprised of two *rap1a* genes only [39,47,48,49]. In a study of five Brazilian *B. bovis* isolates the *rap-1* genes were 98–100% identical to one another and with the T2Bo isolate, suggesting strong sequence and structural conservation [42]. Variability is greater in *B. bigemina*, where there are three distinct *rap1* gene types, *rap1a*, *rap1b*, and *rap1c*. *rap1a* is further subdivided into α1, β1, β2, and β3 isoforms. These are interspersed as five pairs of *rap1a* and *rap1b* genes, followed by a single *rap1c* gene [50]. In *B. gibsoni* and Chinese *B. motasi*-like sheep isolates (*Babesia* sp. BQ1 Lintan) there are also three, with similar organizations as in *B. bigemina* [38,51]. In *B. caballi*, there are at least two *rap1* genes and proteins, with greater diversity among isolates than in other species, complicating the use of an immunodiagnostic based upon this antigen to regulate the movement of horses internationally [52,53]. Similarly, in *B. ovata* there are nine *rap1* genes, with a large degree of variation in length [54]. A comparative sequence study of nine *B. bigemina* isolates from North America, South America, and Puerto Rico revealed very limited sequence diversity. That which was detected allowed identification of markers suggesting long-term stability in gene structure. However, the rare occurrence of unequal recombination and gene conversion reactions among members of the family was also implied [40]. In each sequenced species the *rap-1* genes are present in a locus comprised of tandem repeats. Given the high diversity among species but very limited diversity within a species, the *rap-1* family appears likely to have undergone significant diversification and duplicative expansion subsequent to speciation.

**Figure 2:**
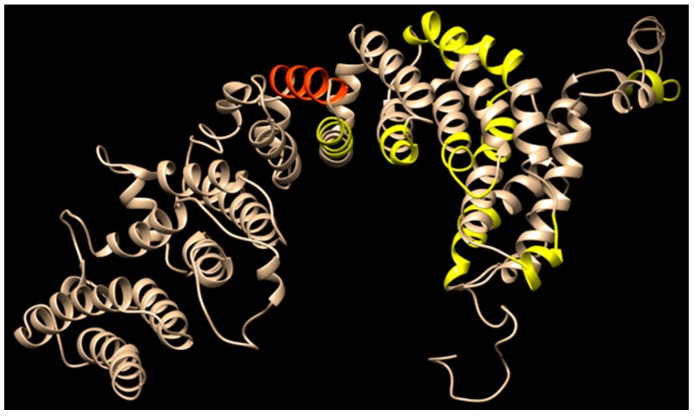
Predicted three-dimensional structure of RAP-1 polypeptide. The three-dimensional structure of RAP-1 was predicted using Robetta [56,57] and displayed with Chimera software [58]. The predicted structure is one of a flattened helix comprised of alternating α-helices in opposing orientations, with an overall curve. The sequences shown in orange are the mapped B-cell epitopes recognized by monoclonal antibodies 23/38.120 and MBOC79, respectively [43], whereas residues colored yellow represent a slightly degenerate form that is repeated eight times, only two copies of which appear to be accessible.

Do RAP-1 proteins contribute to persistence of these parasites? The answer is not obvious. Although immunization with recombinant RAP-1 can generate antibody responses sufficient to interfere with in vitro binding and even invasion, the proportion of merozoites blocked from invading is not more than approximately 60% [34,35]. This suggests the evolution of a protein structure in which B-cell epitopes are not readily accessible on the surface of native protein as it is displayed during invasion, or compete ineffectively with host ligands. Support for this idea comes from mapping conserved B-cell epitopes recognized by monoclonal antibodies 23/38.120 (indicated in orange) and MBOC79 (indicated in yellow) (Figure 2) [43]. Despite the presence of a total of nine degenerate repeats of the MBOC79 epitope, only two are predicted to be exposed on the surface of the RAP-1 polypeptide, whereas the remainder are predicted to be inaccessible (Figure 2). In order to block binding during invasion, the antibody likely must compete for binding with those host proteins bound by RAP-1. Should RAP-1 undergo a conformational change when interacting with the host ligand that renders it no longer susceptible to the antibody, then that competition will generally be won by the host ligand. The result would be an equilibrium favoring the invasion pathway over blocking by the antibody, and thus an inability to efficiently prevent the contribution of RAP-1 to erythrocyte invasion. This dynamic suggests that evolution has selected a structural equilibrium enabling these proteins to retain essential functions even while acting as immunologic smokescreens and in the presence of antibodies. It seems that among species, RAP-1 B-cell epitopes may be conserved but poorly immunogenic, or immunodominant but poorly conserved, but either scenario means there would not be cross-species protection based upon RAP-1 under natural circumstances.

### 2.3. Variant Proteins—VESA1

An unusual aspect of *B. bovis* biology is the ability of infected erythrocytes to cytoadhere to the capillary and post-capillary endothelium, resulting in sequestration of infected erythrocytes within the microvasculature of several major organs [59,60,61]. This behavior was associated with isolate-specific antigenic changes [62,63] that were identified as parasite-derived proteins [13,64]. Upon closer inspection, these proteins were observed to undergo frequent changes in their immunoreactivity during the course of an ongoing infection [14,65]. The development of monoclonal antibodies to one of the variant proteins, referred to as “VESA1a” (variant erythrocyte surface antigen 1a), enabled the identification of the *ves1*α gene encoding this protein. Very significantly, *ves1*α was found to be but one member of a large multigene family, *ves* [15]. VESA1a is normally paired with a second polypeptide, VESA1b, encoded by a *ves1*β gene [13,31,55]. When the sequence of the *B. bovis* genome was obtained, this allowed accurate characterization of nearly the full *ves* multigene family of the T2Bo isolate, defining this family as having more than 104 members, with a preponderance in the *ves1*α branch [4]. A very small third branch of only three copies, *ves1*γ, was observed as well, but is of unknown function. The family has undergone further diversification among *Babesia* spp. into related but distinct *ves2* isoforms, of which *B. bovis* possesses only two [66]. The *ves* gene family is conserved but highly diversified among various *Babesia* spp. sensu stricto, attesting to its significance to the survival and persistence of these piroplasms. Indeed, this family comprises 4.7% of coding sequences in the *B. bovis* genome, 8% in *B. bigemina*, and over 11% in *B. divergens* [66]. The origin of this family is relatively recent. It has been found in some form in all true *Babesia* spp. that have been surveyed at the sequence level [55,66]. However, only three distantly related genes are present in *Babesia microti*, which is not a member of *Babesia* sensu stricto, and the family is either not present or is unrecognizable in *Theileria* spp. Therefore, this gene family appears to have arisen subsequent to the split from the last common ancestor shared with *Theileria* and other piroplasms. However, given that the *ves1*α/*ves1*β branches in *B. bovis* do not appear to be orthologous to the *ves1a*/*ves1b* branches in *B. bigemina*, *B. divergens*, or *B. ovata*, the ancestral *ves* gene from which the family arose likely pre-dates the diversification of modern *Babesia* spp. [55,66]. The *ves* gene duplication, and family branching and expansion in each species are evolutionarily recent events.

In *B. bovis* VESA1 proteins are large in size, being approximately 100 kDa or more in mass, with multiple identifiable domains (Figure 1B) [15,31,55,65]. Following a highly variable N-terminal region, both VESA1a and 1b polypeptides possess a cysteine- and lysine-rich domain (CKRD) which is highly variable among individual paralogs. Despite the high level of variability, highly conserved sequence patches are present, scattered down the length of the gene. Sequences encoding conserved patches flanking the CKRD domain sequences in *ves1*α genes have been used as targets of “universal primers” for PCR amplification, capable of detecting that region in most VESA1a polypeptide transcripts. This approach has revealed the ongoing diversity that occurs there over time, as well as the monoparalogous nature of *ves* gene transcription [67,68]. Following the CKRD domain is a stretch of a relatively disordered sequence that is quite variable among gene copies and lacking in any notable conserved motifs. Embedded within this disorder is the nearly invariant motif, P(K/R)TIREILYWLSALPYS, that effectively identifies any paralog of either branch within *B. bovis*. Degenerate forms of this motif are found in *ves1* genes of other *Babesia* sensu stricto as well. The C-terminal to the disordered region is a “variant domain conserved sequences” (VDCS) region in VESA1a polypeptides. Like the CKRD, the VDCS domain is relatively rich in cysteine and lysine residues, and was given its conflicted name due to the presence of multiple conserved sequence motifs embedded within highly variant sequences. The positioning of these motifs allows division of the VDCS into two subdomains, VDCS1 and VDCS2, each defined by a unique motif embedded within variant sequences. Importantly, the VDCS region partially overlaps a paired coiled-coil domain that may play a role in subunit interactions during assembly of the holoprotein. Instead of a VDCS, VESA1b polypeptides possess a “low complexity variant domain” (LCV) lacking any notable motifs. The VDCS/ LCV regions of both classes are followed by a transmembrane domain that anchors the polypeptide within the host erythrocyte membrane and a short, relatively conserved C-terminus that resides within the erythrocyte cytoplasm [15,31]. Both the CKRD and VDCS domains are lacking from VESA proteins encoded by *ves1*a and *ves*1b genes in *B. bigemina*, *B. divergens*, and *B. ovata* [54,66], none of which cytoadhere or form the ridge-like structures found on *B. bovis*-infected erythrocytes.

**Figure 3:**
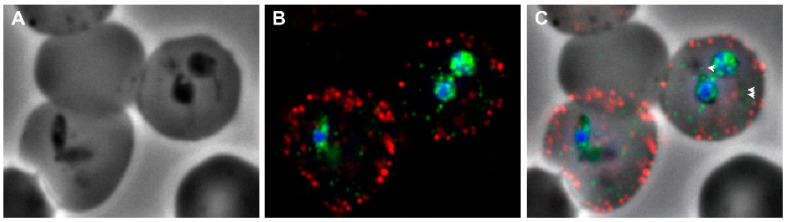
Immunolocalization of VESA1a and SmORF in *Babesia bovis*-infected erythrocytes. (**A**) *B. bovis*-infected bovine erythrocytes are shown by phase contrast, revealing paired merozoites. (**B**) Immunofluorescence was used to localize VESA1a proteins with monoclonal antibody 4D9.1G1 (red), and a model SmORF-GFP fusion protein with a rabbit anti-GFP antibody (green). Parasite nuclei are revealed by counterstaining with DAPI (blue). (**C**) Overlay of immunofluorescence and phase-contrast images to enable visualization of label relative to structures. Intensity ranges have been adjusted (uniformly) to facilitate visualization of faint fluorescence signals arising from within the erythrocyte cytoplasm. The separate movement of both proteins through the infected erythrocyte cytoplasm as small “packets”, many associated with vesicular structures (examples identified with arrowheads), is easily seen (described in [37]).

As with a number of protozoal parasite proteins exported to the host cell, there is no N-terminal or cleaved signal peptide for export. Presumably, an internal hydrophobic patch acts as an uncleaved intramolecular signal for membrane traversal [15,31,66]. The means by which this protein is trafficked to the erythrocyte membrane is unknown, but a clue may have been found during characterization of transcription in attenuated *B. bovis* strains. Parasites attenuated by rapid passage through splenectomized calves consistently up-regulate the *sbp2t11* gene encoding a truncated spherical body protein [69]. Up-regulation of the SBP2t11 protein results in decreased cytoadhesion of infected erythrocytes [70]. Since VESA1 protein(s) are major players in *B. bovis* cytoadhesion, SBP2t11 overexpression may disrupt the normal trafficking of VESA1 polypeptides to the erythrocyte membrane, suggesting the possible involvement of the spherical body compartment in the export pathway. Evidence to date indicates that proteins targeted to *B. bovis* spherical bodies appear to use a cleaved N-terminal signal sequence and a second, cleaved trafficking motif, RXXL or RXLX, related to the “PEXEL” motif found in *Plasmodium* spp. [37]. Similar conserved motifs are found within most VESA1 polypeptides, but the motifs are embedded deep within the proteins and the proteins are not post-translationally cleaved [15]. The question of how VESA polypeptides are trafficked to the erythrocyte membrane warrants further investigation, although once within the infected erythrocyte cytoplasm they appear to be trafficked in small boluses distinct from those of a model SmORF protein (Figure 3). Detailed knowledge of this process potentially could provide a novel strategy by which to control this group of parasites.

There is no question that VESA proteins contribute directly to the survival and persistence of babesial parasites. In *B. bovis* VESA1 proteins are intimately involved in cytoadhesion [71,72]. As a result of its cytoadhesive behavior, circulating parasitemias in *B. bovis* infections do not accurately reflect actual parasite burdens and adversely affect the sensitivity of both microscopic and molecular diagnostic tests. This behavior also means these microaerophilic parasites spend a large portion of their asexual reproductive cycle within a hypoxic environment, sheltered from the oxidative damage inherent to life within a red blood cell [73]. An added benefit of sequestration in the microvasculature is an opportunity to forego passage through the spleen, where most infected erythrocytes might be subject to clearance simply due to the metabolic and structural alterations they cause the host cell [74]. The significance of parasite sequestration to the host is not clear, with different studies providing conflicting evidence for a contribution to pathology [59,75,76,77,78,79,80]. It is likely that cytoadhesion acts largely as a triggering event, and localized physiological responses cause the real pathology [81]. Significantly, antibodies directed to VESA1a (and probably VESA1b, although this has not been established) are capable of blocking cytoadhesion and even reversing existing cytoadhesive interactions in vitro [72]. In vivo, where hydrodynamic shear forces generated by blood flow also must be dealt with, the effects of antibody binding are likely to be even greater. This may explain why *B. bovis* has evolved to cluster VESA1 proteins over the ridge-like structures it forms in the infected erythrocyte membrane, as this could facilitate higher avidity cooperative interactions [72]. Indeed, in the virulent human malaria parasite, *P. falciparum*, disruption of the ability to form similar knob-like structures where its PfEMP1 cytoadhesion ligand clusters in the infected erythrocyte membrane interferes with that parasite’s ability to cytoadhere under shear, but not under static conditions [82].

**Figure 4:**
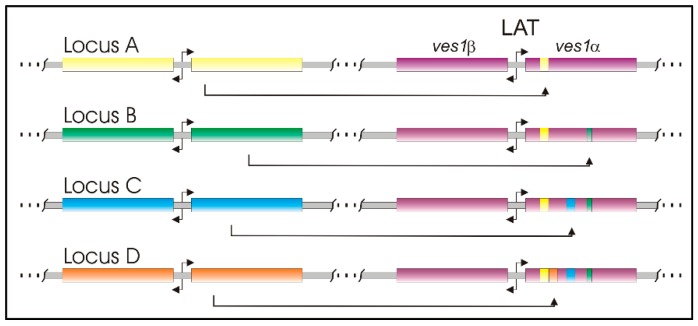
Segmental gene conversion of *ves* genes. Much of the *ves* gene family is organized as pairs of genes apposing single, bidirectional promoters (bent arrows). Transcription of *ves* genes occurs from a single locus at a time, which is referred to as the “Locus of active *ves* transcription” (LAT), although there are numerous silent *ves* loci with the potential to become an LAT through in situ switching. The *ves1*α and *ves1*β genes within the LAT are both progressively modified over time by SGC, a replacement of sequences by a gene conversion-like process involving short segments copied from silent *ves* loci (shown only for the *ves1*α gene). This is indicated by colored patches derived from silent loci that remain unchanged by the process of donation. The donor locus may be on the same or any of the other three nuclear chromosomes.

In order to protect the VESA1 cytoadhesion function from abrogation by antibodies, *B. bovis* has evolved the ability to rapidly mutate the expressed isoform, a process referred to as antigenic variation. Among eukaryotic systems that have been studied, antigenic variation sensu stricto involves multigene families devoted to, or at least specialized for, this rapid mutation [83]. In most instances, only one or a small number of paralogs are transcribed at one time by individual parasites [20]. Approximately 70% of the *ves* genes encoding VESA1 polypeptides, of which there are more than 100, are arranged as divergently oriented pairs (Figure 4). These pairs may be comprised of one *ves1*α and one *ves1*β gene, or two *ves1*α genes [4,31]. Transcription of *ves* genes generally involves two *ves* gene copies, one *ves1*α and one *ves1*β gene, but a single locus of active *ves* transcription (LAT) [31]. However, transcription appears to be monoparalogous with respect to members of a particular branch [31,67,68]. This is logical, as the VESA1 holoprotein is a heterodimer comprised of VESA1a and 1b polypeptides [13,14], and the genes in a *ves* gene pair share a single bidirectional promoter driving expression of the two subunits [84]. Variation in the structure of VESA1 has been demonstrated to occur through a process of segmental gene conversion (SGC) [31]. In SGC the actively transcribed *ves* genes are progressively modified by replacement of short sequence patches with sequences from non-transcribed donor genes, typically without affecting the donor. SGC occurs in both transcribed *ves* genes, leading to formation of genes (and proteins) that are mosaics of the original and donor genes (Figure 4) [31]. The result is that great variation can be generated quickly in the individual subunits, and since variation in the holoprotein is the product of that of the subunits the total variation is extreme. In our personal experiences attempting to raise antibodies against recombinant polypeptides comprised of conserved sequences within the VESA1b polypeptide, the immune system responded only to the variant sequences [55].

It is easy to envision how rapid antigenic variation of *ves* genes shields VESA1 polypeptides from immune recognition and loss of function. Harder to envision is how these proteins acquire an adhesive function in the midst of all this structural change. There are generally no specific domains predicted within VESA1 polypeptides that resemble known binding domains at the sequence level. On the other hand, the large variety of sequence combinations engendered by SGC may be key. With the stochastic creation of a large repertoire of unique sequence variants by SGC, coupled with the innate flexibility of polypeptides, and stabilized by disulfide bonds formed between cysteine residues in the cysteine-rich domains, VESA1 polypeptides can likely be formed that would interact with a wide variety of ligands. This strategy could be thought of as analogous to random peptide phage display or aptamer selection (or an imaginative child with LEGO® toys). Whereas this would not seem to be a good strategy for deliberately forming variant binding proteins that could interact with a specific receptor (the evolutionary path clearly taken by *P. falciparum* with its PfEMP1 cytoadhesion ligand), this may allow the formation of VESA1 proteins that can bind to almost any target. VESA1 isoforms that bind productively would result in sequestration of the parasite expressing them, and selection of that parasite for survival and population expansion. It should also be noted that, many sequence combinations would likely not result in a functionally adhesive molecule. Those parasites expressing such non-functional VESA1 isoforms would be strongly selected against. However, with the immense numbers of parasites present, even at low parasitemias, the variety of possible molecular surfaces that could be presented and the “chemical space” that could be sampled in this way would be very great. Although direct experimental proof of such a strategy has not been developed, this idea is consistent with both SGC modification of these proteins and the observation that *B. bovis* may sequester in a very wide variety of tissues, the identity of which may change over time [59,60,61]. Thus, although we do not yet know how VESA1 mediates cytoadhesion, it is possible to hypothesize a plausible, testable mechanism despite extreme variation.

### 2.4. Protein Families of Uncertain Behavior—SmORF

The *smorf* genes (for small open reading frame) form a relatively large multigene family of 44 members that is found only in *B. bovis*. This family, the second largest in *B. bovis*, is divided into two branches, A and B. The *smorf* multigene family was a surprise discovery upon sequencing of the *B. bovis* genome, where members of this family were found to be interspersed among *ves* genes, embedded within *ves* gene clusters [4]. The reason for this close proximity with *ves* genes is unclear, as the two families do not appear to be related genetically. One early suggestion was that proximal positioning could serve to regulate coordinate transcription of the two gene families, but there is no evidence currently in support of such coordination. In fact, *smorf* transcription may be much more promiscuous and less regulated than is *ves* transcription. In contrast with the monoparalogous transcription of *ves* genes, as many as 32 of the 44 *smorf* genes reportedly were transcribed by the parasite population found within deep brain tissues of a calf that died of neurologic complications during acute infection [16]. However, there are multiple caveats to this observation: (i) the infection was initiated with a non-clonal *B. bovis* isolate, T2Bo; (ii) the brain tissue used for analysis was packed with sequestered parasites, possibly of differing VESA1 phenotypes transcribed from different active *ves* loci; and (iii) transcription was assayed by a non-quantitative end-point RT-PCR, with cloning and sequencing of the products. Because of these caveats it remains unclear whether transcription is perhaps monoparalogous as it is with *ves* genes, and multiple *smorf* genes are detected because individuals within different segments of the population are transcribing single, different genes, or whether individual parasites really are transcribing multiple different *smorf* genes simultaneously at significant levels. If individual parasites are transcribing multiple different *smorf* genes simultaneously, this brings into question how *smorf* genes escape the (presumably) epigenetic silencing bestowed upon their closely neighboring *ves* genes. Thus, this remains an open question.

SmORF proteins have a repetitive domain structure that enables their classification into types A and B (Figure 1C). Both types possess an N-terminal, cleaved signal peptide for entry into the endoplasmic reticulum, followed by a low-complexity region with a short embedded hypervariable region (HVR). A domain follows that is comprised of a degenerate type A or B repeat, a low-complexity region, and a C-terminal HVR. This pattern defines the complete structure of type B SmORF proteins. Type A SmORF proteins have, in addition, a second type A repeat C-terminal to the first, with a large HVR separating the two repeats [16]. In addition to these domains, SmORF proteins also possess an internal, proteolytically cleaved trafficking signal, RXXL, near the N-terminal end of the initial repeat domain that is analogous to the “PEXEL” motif of malarial parasites. In *Plasmodium* spp. cleavage of the PEXEL is required to “license” certain proteins for export beyond the parasitophorous vacuolar membrane [85,86,87]. *Babesia* spp. do not maintain a parasitophorous vacuole membrane like *Plasmodium*, eliminating it very rapidly following erythrocyte invasion [88]. However, processing of the PEXEL-like signal is important for retention of mature type B SmORF proteins within the spherical body compartment. From the spherical bodies a proportion is released into the infected erythrocyte in which the parasite resides (Figure 3), but the bulk is released during the next round of erythrocyte invasion by the merozoite [37]. It has not yet been determined experimentally whether this also holds for type A SmORF proteins. It should be noted that both repeat domains in type A SmORF proteins contain a conserved RXXL trafficking motif. The extent of processing to which type A SmORF proteins are exposed has not been determined. However, that this gene family appears to have undergone expansion subsequent to domain duplication, and yet has retained this signal in the duplicated domain suggests that the presence and processing of the PEXEL-like motif serves an essential purpose.

Do SmORF proteins have an impact upon the persistence of *B. bovis*? No definitive answer is available at this time, but this is likely the case. Using the spherical body protein-2 (SBP2) family as a comparative example, a mechanism was suggested by which up-regulation of Sbp2t11 expression may alter protein trafficking to effect attenuation [69,70,89]. Both SBP2 and SmORF proteins traffic to the host erythrocyte by way of the spherical body compartment [12,90], with SmORF proteins being staged there for control of subsequent release [37]. Should SmORF protein levels impact the trafficking of other proteins similarly to Sbp2t11, then changes in the types, numbers, and levels of SmORF proteins expressed could also affect the expression or function of host-parasite interaction proteins, analogous to phase variation in bacteria. Since SmORF proteins must be processed for proper localization [37], even the levels of expression of the responsible protease could impact on the situation, affecting both trafficking and the forms in which SmORF and perhaps other proteins arrive at their destinations. There is at present no direct evidence for such an effect, but these are testable ideas. The fact that SmORF proteins are unique to *B. bovis* and encoded by an amplified gene family suggests this protein family has evolved to play a specific, significant role in the biology of this parasite.

## 3. Conclusions

There are a great many aspects of the persistence of babesial organisms in the vertebrate host that we do not fully understand. In this review an attempt was made to demonstrate how variable and variant proteins are similar and how they differ, and how those distinctions may affect the nature of their contributions to parasite persistence. Along with well-established observations there is a good deal of conjecture in this review. The intention was to stimulate novel ways to think about the contributions of protein structure to the capacity of parasites for survival, using *B. bovis* as the focal point. What is abundantly clear is that the interplay between host and pathogen is quite complex, and this has strongly impacted the evolution of parasite genomes.

## Figures and Tables

**Table 1:** General characteristics of variable and variant multigene families.

Variable Families	Variant Families
Usually two to several members	Families are generally large (>20 members)
>One member may be expressed at a time	Expression monoallelic or monoparalogous
Expressed gene(s) stable over course of infection	Expressed gene altered often during infection
Recombination infrequent and incidental	Recombination common and family-targeted
Family diversity relatively stable among isolates	Family diversity less stable among isolates
